# Antimicrobial Activity of *Cinnamomum cassia* Essential Oil: Phytochemical Profiling, In Vitro Evaluation, and Molecular Docking

**DOI:** 10.1002/cbdv.71767

**Published:** 2026-09-27

**Authors:** Asma Bouguerra, Asma Meziti, Hassina Guergour, Naouel Boussoualim, Imane Krache, Nadira Oukala, Amal Saidi, Daoud Harzallah, Shakilur Rahman, Hamdi Bendif, Gharieb S. El‐Sayyad, Stefania Garzoli

**Affiliations:** ^1^ Laboratory of Applied Microbiology, Faculty of Natural and Life Sciences Ferhat Abbas Setif 1 University Setif Algeria; ^2^ Department of Biology, Faculty of Natural and Life Sciences / Earth and Universe Sciences Mohamed El Bachir El Ibrahimi University Bordj Bou Arreridj Algeria; ^3^ Laboratory of Health and Environment, Faculty of Natural and Life Sciences / Earth and Universe Sciences Mohamed El Bachir El Ibrahimi University Bordj Bou Arreridj Algeria; ^4^ Laboratory of Applied Biochemistry, Department of Microbiology, Faculty of Nature and Life Sciences University Ferhat Abbas Setif 1 Setif Algeria; ^5^ Laboratory of Applied Biochemistry, Department of Biochemistry, Faculty of Nature and Life Sciences University Ferhat Abbas Setif 1 Setif Algeria; ^6^ Laboratory For the Valorization and Conservation of Biological Resources, Department of Biology M'hamed Bougara University Boumerdes Algeria; ^7^ Department of Biology, College of Science Imam Mohammad Ibn Saud Islamic University (IMSIU) Riyadh Saudi Arabia; ^8^ Department of Chemistry and Technologies of Drug Sapienza University P. Le Aldo Moro Rome Italy

**Keywords:** (E)‐cinnamaldehyde, molecular docking, antifungal activity, *Cinnamomum cassia* essential oil, GC–MS analysis, antibacterial activity, minimum inhibitory concentration

## Abstract

In this study, *Cinnamomum cassia* bark essential oil (CEO) was extracted with a yield of 2.51% and characterised by its organoleptic properties, physicochemical characteristics, and gas chromatography–mass spectrometry (GC–MS) profiling. Seven phytoconstituents were identified, with (E)‐cinnamaldehyde predominating (87.14%). CEO inhibited *Staphylococcus aureus* ATCC 25923*, Bacillus subtilis* ATCC 6633, *Escherichia coli* ATCC 25922, and *Pseudomonas aeruginosa* ATCC 27853, with inhibition zones of 16.0 ± 1.0–47.0 ± 1.0 mm. Minimum inhibitory concentration (MIC) and minimum bactericidal concentration (MBC) values ranged from 0.52–1.04 and 0.52–2.08 mg/mL, respectively, with greater susceptibility observed among the gram‐positive strains. Antifungal evaluation revealed potent activity against *Alternaria alternata* and moderate activity against *Aspergillus niger*, with direct‐contact exposure showing greater inhibition of *As. niger* than vapour‐phase treatment. Molecular docking identified coumarin, a minor constituent, as exhibiting the strongest predicted binding, with binding energies of −7.7 kcal/mol against the bacterial targets TEM‐1 β‐lactamase and D‐alanine:D‐alanine ligase, and −7.0 kcal/mol against tyrosyl‐tRNA synthetase. Coumarin also showed favorable predicted binding to the fungal targets cytochrome P450 51B (CYP51B) and epoxide hydrolase. These findings support the potential of CEO as a natural antimicrobial preservative, although further validation in real food systems is required.

## Graphical abstract

Phytochemistry, bioactivity, and molecular docking of *Cinnamomum cassia* EO.

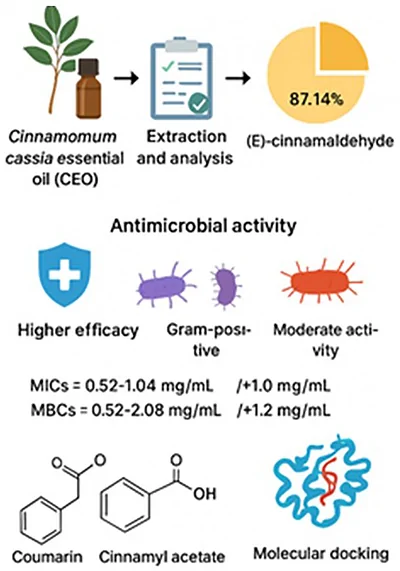

## 1 Introduction

Unsafe food remains a major global public health threat, resulting in millions of cases of foodborne illness and hundreds of thousands of deaths each year. Children are often at greatest risk and constitute a substantial fraction of these cases [1]. As a result, food safety has become one of the most significant priorities in public health strategies worldwide. Despite ongoing control measures, recurrent outbreaks caused by pathogenic and spoilage microorganisms continue to compromise the safety, quality, and stability of the global food supply [2]. This challenge is further compounded by the emergence of multidrug‐resistant foodborne pathogens, which can substantially reduce the effectiveness of conventional control measures [3].

One of the primary strategies for mitigating these risks is the use of food preservatives. These substances, either naturally present in foods or added to them, inhibit microbial growth, prevent lipid oxidation, and extend the shelf life of foods [4]. However, synthetic preservatives such as nitrates, benzoates, sulfites, parabens, and formaldehyde have been linked to various health risks, including hypersensitivity, allergies, asthma, hyperactivity, neurological damage, and cancer. This has fueled the growing preference for safer and more natural alternatives [5]. Consequently, both the food industry and scientific community have shifted their focus toward sustainable, bio‐based alternatives to satisfy the growing global demand for clean‐label food products [6].

**FIGURE 1:**
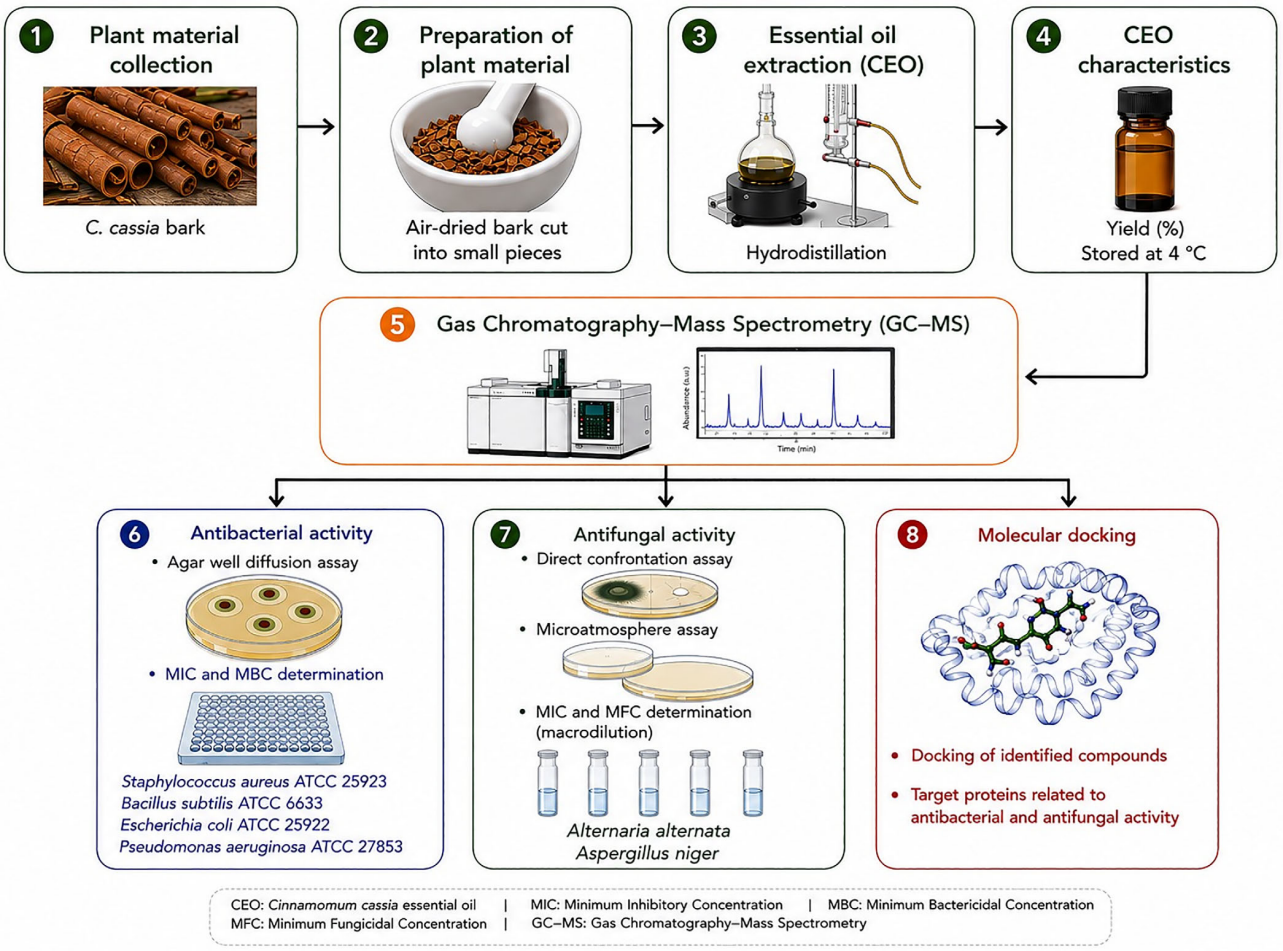
Overall experimental workflow of the study.

In this context, essential oils have emerged as promising natural preservatives in food products. These volatile aromatic compounds are extracted from different parts of plants, including flowers, leaves, seeds, peels, bark, wood, roots, and rhizomes [7, 8]. They contain a complex mixture of bioactive compounds, such as terpenes (monoterpenes, sesquiterpenes, and diterpenes), terpenoids, phenolics, and aldehydes, each of which plays a crucial role in their biological and therapeutic potential [9, 10]. Among the numerous essential oils studied, *Cinnamomum cassia* essential oil (CEO) (Lauraceae family) has garnered considerable attention for its strong antimicrobial activity against foodborne pathogens and fungi, as well as its notable anti‐inflammatory and antioxidant properties. These biological activities are primarily attributed to its rich composition of bioactive constituents, including cinnamaldehyde, β‐pinene, and cinnamic acid [11, 12]. In addition to its therapeutic potential, cinnamon is widely used in seasonings, sauces, baked goods, confections, and beverages and has been recognized by the Food and Drug Administration as a safe food additive [13]. To further explore the potential molecular basis of these antimicrobial effects, molecular docking can provide insights into the interactions of essential oil constituents with relevant microbial targets [14, 15]. Although *Cinnamomum cassia* essential oil (CEO) has been widely studied for its antimicrobial properties, the integration of phytochemical characterisation, experimental antimicrobial assessment, and molecular docking remains limited. This study therefore aimed to characterize the chemical composition of CEO and evaluate its antibacterial and antifungal activities, complemented by molecular docking analysis to predict the potential interactions of its major and minor constituents with selected microbial targets. The schematic workflow of this study is illustrated in Figure 1.

## 2 Material and Methods

### 2.1 Extraction of CEO

The bark of *Cinnamomum cassia* was purchased from a local medicinal herb market in Bordj Bou Arreridj, Algeria, in March 2024. The plant material was botanically identified by Prof. Wafa Nouioua, a specialist in plant identification in the Department of Ecology, University of Setif 1. A voucher specimen was registered under WFO‐0001285954.

For essential oil extraction, approximately 150 g of dried bark was cut into small pieces and subjected to hydrodistillation using a clevenger‐type apparatus. The bark pieces were placed in a flask containing 750 mL of distilled water and gently boiled for 4 h. During distillation, steam carried the volatile compounds through the condenser, where they cooled and formed a mixture of oil and water. The essential oil was then carefully separated, dried over anhydrous sodium sulfate, and stored in a tightly sealed amber glass bottle at 4°C until further analysis [16].

### 2.2 Organoleptic and Physicochemical Characteristics of CEO

The organoleptic and physicochemical properties of CEO were determined according to standard analytical procedures. Organoleptic characteristics, including color, odor, and appearance were assessed by visual and olfactory examination. Physicochemical parameters, such as yield, density, and refractive index were measured to assess the quality and purity of the essential oil [17].

### 2.3 Chromatography–Mass Spectrometry (GC–MS) Analysis of CEO

The volatile components of CEO were examined using gas chromatography (Agilent 8890) coupled with a mass spectrometer detector (Agilent 5977B) equipped with a DB‐5MS capillary column (60 m × 250 µm i.d. × 0.25 µm film thickness), selected to ensure high‐resolution separation of the volatile constituents Helium gas, which served as the carrier gas was maintained at a constant pressure of 65 kPa. A solvent delay of 4 min was used with an injection of 1 µL of essential oil in a split ratio of 1:50. The oven temperature was initially set to 50°C and increased to 240°C at a rate of 5°C/min. The compounds were identified by comparing their mass spectral fragmentation patterns with those reported in the database library (NIST2020.L). Quantitative determination was based on the integration of peak areas [18].

### 2.4 Antibacterial Activity of CEO

To evaluate the antibacterial activity of CEO against gram‐positive and gram‐negative bacterial strains, the agar well diffusion assay was performed as a preliminary screening method using four reference bacterial strains: *Bacillus subtilis* ATCC 6633, *Staphylococcus aureus* ATCC 25923, *Escherichia coli* ATCC 25922, and *Pseudomonas aeruginosa* ATCC 27853. All strains were obtained from the American Type Culture Collection (ATCC). The assay was performed following the procedure described by Elgammal et al. [19], with minor modifications. CEO was diluted in dimethyl sulfoxide (DMSO) to obtain concentrations of 10%, 5%, 2.5%, and 1.25% (v/v). Bacterial suspensions were adjusted to a turbidity equivalent to 0.5 McFarland (≈1.5 × 10^8^ CFU/mL), and Mueller–Hinton (MH) agar plates were uniformly inoculated. Wells with a diameter of 6 mm were filled with 50 µL of each CEO dilution, and DMSO served as a negative control. The plates were incubated aerobically at 37°C for 24 h. After incubation, inhibition zones were measured in millimeters and reported as mean ± standard deviation (SD) (*n* = 3).

### 2.5 Minimum Inhibitory Concentration (MIC) and Minimum Bactericidal Concentration (MBC) of CEO

To determine the MIC and MBC values, the broth microdilution assay was conducted according to Zhang et al. [2], with minor modifications. Briefly, two‐fold serial dilutions of CEO were prepared in 96‐well microplates containing 50 µL of Mueller–Hinton (MH) broth per well to obtain final CEO concentrations ranging from 16.64 to 0.13 mg/mL after addition of the bacterial inoculum. Subsequently, 50 µL of bacterial suspension adjusted to 0.5 McFarland and diluted to approximately 5 × 10^5^ CFU/mL was added to each well. Growth control wells containing MH broth and bacterial inoculum without CEO, solvent control wells containing MH broth, Tween 80, and bacterial inoculum without CEO, and sterility control wells containing MH broth alone were included in each assay. The microplates were incubated at 37°C for 24 h. The MIC was defined as the lowest CEO concentration that completely inhibited visible bacterial growth.

To determine the MBC, 10 µL aliquots from wells showing no visible growth were subcultured onto nutrient agar plates and incubated at 37°C for 24 h. The lowest concentration that produced no bacterial colonies was recorded as the MBC. The assay was performed in triplicate.

### 2.6 Antifungal Activity of CEO

The antifungal activity of CEO was evaluated against *Alternaria alternata* and *Aspergillus niger*, both isolated from decayed fruits. The direct confrontation method was used to assess the antifungal effect of CEO under contact conditions, whereas the microatmosphere assay was used to evaluate the inhibitory effect of CEO volatiles in the absence of direct contact with the fungal mycelium.

#### 2.6.1 Direct Confrontation Method

The antifungal activity of CEO was assessed following the methods described by Maluleke et al. and Nacef et al. [20, 21]. Mycelial discs (6 mm in diameter) were excised from 7‐day‐old fungal cultures and placed at the center of Potato Dextrose Agar (PDA) plates. At a distance of approximately 40 mm from the inoculum, 6 mm Whatman No. 1 filter paper discs impregnated with 20 µL of CEO were positioned. Control plates were prepared under identical conditions using sterile distilled water instead of CEO. The plates were incubated at 28°C for seven days. The percentage of fungal growth inhibition (I%) was calculated using the following equation: I (%) = [(*D*—Di)/*D*] × 100 where *D* represents the mycelial growth diameter in the control plates and Di corresponds to the mycelial growth diameter in the presence of CEO. The assay was performed in triplicate.

#### 2.6.2 Microatmosphere Assay

The antifungal activity of CEO volatiles was evaluated using a microatmosphere assay. Following the methods described by Soković et al. and Anjum and Akhtar [22, 23], mycelial discs (6 mm in diameter) were excised from 7‐day‐old cultures and placed at the centre of Petri dishes containing Potato Dextrose Agar (PDA). Sterile Whatman filter paper discs (6 mm in diameter) were impregnated with 20 µL of CEO and affixed to the inner surface of the Petri dish lid. The dishes were inverted and sealed with Parafilm to minimize vapor loss and ensure uniform diffusion of volatile compounds. The plates were incubated at 28 °C for seven days, after which the mycelial growth diameter was measured. The assay was performed in triplicate. The percentage of mycelial growth inhibition (I%) was calculated using the following equation: I (%) = [(*D*—Di)/*D*] × 100 where *D* represents the mycelial growth diameter in the control plates and *Di* corresponds to the mycelial growth diameter in the presence of CEO.

#### 2.6.3 Minimum Inhibitory and Fungicidal Concentrations (MFC) of CEO

Minimum inhibitory concentration (MIC) and MFC determinations were performed to further characterize the inhibitory and fungicidal effects of the oil and performed according to the method described by Doudi et al. [24]. Serial dilutions of CEO (0.5–128 µL/mL) were prepared in tubes containing 1 mL of Potato Dextrose Broth (PDB) supplemented with Tween 20. Each tube was inoculated with 1 mL of fungal suspension containing approximately 10^6^ spores/mL. The tubes were incubated at 28°C for three days. The MIC was defined as the lowest CEO concentration showing no visible fungal growth. For MFC determination, 100 µL aliquots from tubes showing no visible growth were subcultured onto PDA plates. The plates were incubated at 28°C for seven days. The MFC was defined as the lowest CEO concentration that resulted in no fungal growth after subculturing. A growth control containing the fungal inoculum and PDB supplemented with Tween 20 but without CEO was included to confirm normal fungal growth. A sterility control containing PDB supplemented with Tween 20 without fungal inoculum was included to verify the sterility of the culture medium. The assay was performed in triplicate.

### 2.7 Molecular Docking Study

Molecular docking was performed as a predictive in silico approach to investigate the potential interactions of the seven phytochemicals identified in CEO with selected bacterial and fungal target proteins. The analysis aimed to predict ligand binding poses and estimate relative binding affinities, thereby identifying plausible molecular interactions that may contribute to the antimicrobial activity observed experimentally. Simulations were performed using AutoDock Vina program. The selected bacterial target proteins were retrieved from the Protein Data Bank (PDB). For antibacterial activity, the selected proteins represent key enzymatic systems involved in essential bacterial metabolic pathways [25, 26].

These included targets associated with cell wall synthesis, DNA and nucleotide synthesis, and protein synthesis. For antifungal activity, epoxide hydrolase (EH) from *Aspergillus niger* (PDB: 3G0I) and sterol 14‐alpha demethylase (CYP51B; PDB: 5FRB) were selected based on their involvement in fungal metabolic and membrane‐associated processes [27].

The docking grids were centered on the native ligand coordinates to encompass the active sites with dimensions of 40 × 40 × 40 Å. To ensure an effective simulation, the ligands were initially prepared by optimizing their structures using Chem3D program to obtain stable geometries with minimum energy. Next, the receptors (proteins) were prepared using Discovery Studio. This involved removing water molecules and heteroatoms, and adding polar hydrogen atoms and Kollman charges. Afterwards, molecular docking simulations were conducted using the AutoDockVina program. Docking free energies (Δ*G*) were converted into estimated inhibition constants (Ki) using Ki = exp (ΔG/RT) where (R is the gas constant (1.985 × 10^−3^ kcal mol^−1^ K^−1^), and T is the temperature in kelvins (298.15 K) [28]. The calculated binding energies and Ki values should therefore be interpreted as computational estimates rather than experimentally determined inhibition constants.

### 2.8 Statistical Analysis

All assays were conducted in triplicate (*n* = 3) to ensure reproducibility, and data are reported as means ± SD. Antibacterial activity data were analyzed using one‐way analysis of variance (ANOVA) in R software, followed by Tukey's post hoc test for multiple comparisons. Differences were considered statistically significant at *p *< 0.05.

## 3 Results and Discussion

### 3.1 Organoleptic and Physicochemical Profile

The essential oil extracted from *Cinnamomum cassia* bark was a clear yellow to deep yellow liquid, exhibiting the characteristic fresh, spicy aroma of cinnamon [29]. The yield, with an extraction efficiency of 2.51%, aligns with values previously reported for this species [30, 31]. Comparatively, this yield is higher than those typically reported for *Cinnamomum zeylanicum* bark oil, which generally range from 0.5% to 1.2%, indicating a relatively high oil yield for the selected *C. cassia* bark source [32]. The refractive index of the extracted oil, measured at 20 °C using a refractometer, was 1.61. According to the sixth edition of the French Pharmacopoeia, the refractive index of CEO should fall within the range of 1.600–1.614, indicating compliance with the reported physicochemical specification. The density of *Cinnamomum cassia* essential oil ranged from 1.06 to 1.07 g/cm^3^ at 20°C. This value was higher than that reported for *C. zeylanicum* oil (1.019–1.026 g/cm^3^) [33].

### 3.2 Chemical Analysis

**FIGURE 2:**
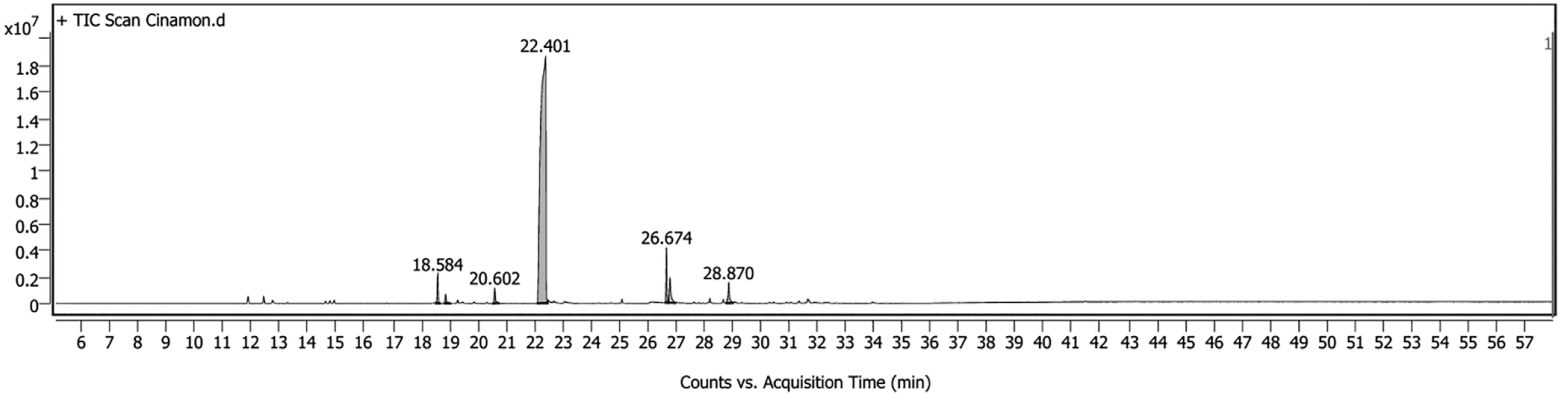
GC–MS chromatogram of *Cinnamomum cassia* essential oil, showing the major compounds identified in the essential oil.

The chemical profile of CEO was analyzed using GC‐MS. The results indicated the presence of seven components (Table 1). All compounds were detected at retention times ranging from 18.58 to 28.87 min (Figure 2). The number of components identified in the essential oil in this study was lower than that reported in some previous studies [13, 34].

**TABLE 1:** Chemical composition of *Cinnamomum cassia* essential oil identified by GC–MS analysis.

No	RT^a^ (min)	Component name	Area %	MW^b^ (g/mol)	MF^c^	Chemical structure
1	18.584	(+)‐2‐Bornanone	1.91	152.23	C_10_H1_6_O	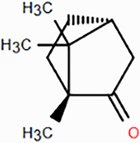
2	18.863	Hydrocinnamaldehyde	0.77	134.18	C_9_H_10_O	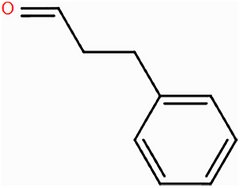
3	20.602	(Z)‐Cinnamaldehyde	1.26	132.16	C_9_H_8_O	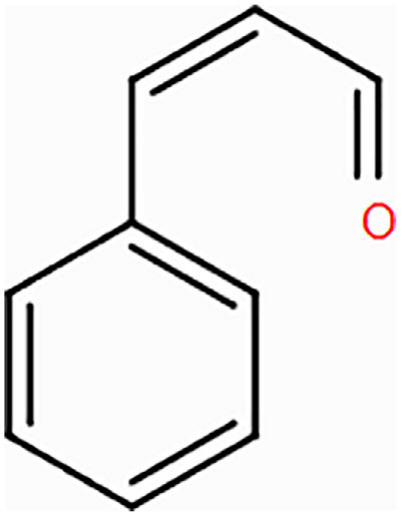
4	22.401	(E)‐Cinnamaldehyde	87.14	132.16	C_9_H_8_O	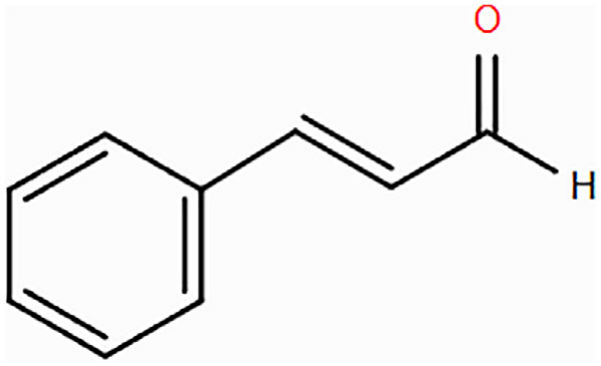
5	26.674	Cinnamyl acetate	3.87	176.21	C_11_H1_2_O_2_	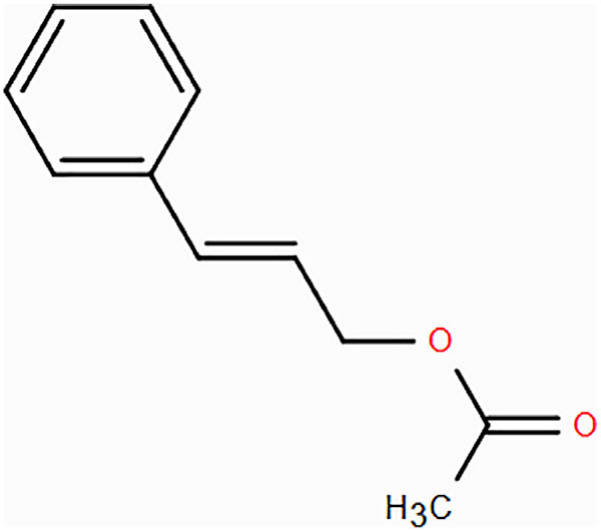
6	26.793	Coumarin	2.84	146.15	C_9_H_6_O_2_	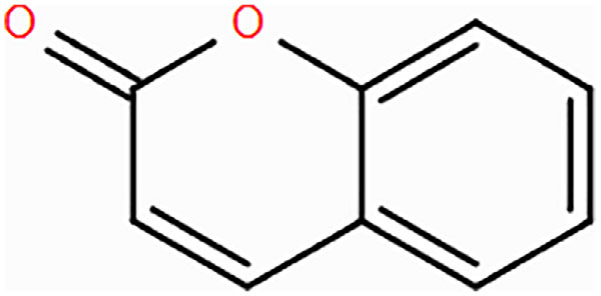
7	28.87	(Z)‐2‐Methoxycinnamaldehyde	2.2	162.18	C_10_H_10_O_2_	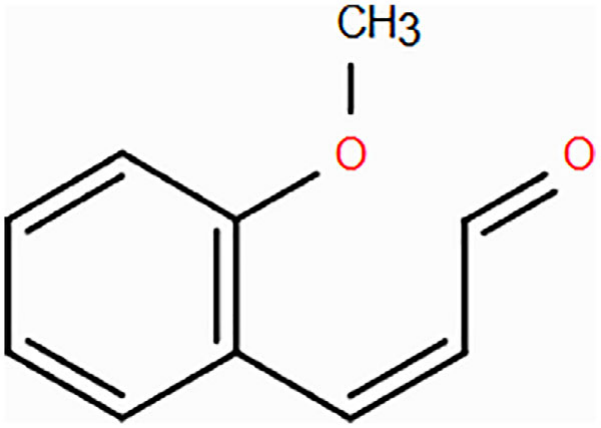

^a^ Retention Time.

^b^ Molecular Weight.

^c^ Molecular Formula.

The GC–MS chromatogram revealed that (E)‐cinnamaldehyde was the predominant constituent, accounting for 87.14% of the total area. This finding is consistent with the characteristic chemical profile of CEO, where (E)‐cinnamaldehyde, a *trans*‐aromatic aldehyde, is responsible for the characteristic spicy, sweet, and slightly pungent scent that defines cassia's sensory profile and bioactive properties [35, 36]. This high proportion is consistent with previously reported data and is characteristic of cassia oil [13, 37].

In addition to cinnamaldehyde, several minor constituents were identified at lower concentrations. Cinnamyl acetate, present at 3.87% is an ester compound that contributes smooth, sweet, and somewhat fruity and floral notes to the oil, thereby balancing the sharper aldehydic character of cinnamaldehyde [13, 37]. Coumarin, detected at 2.84%, is a naturally occurring lactone that imparts a sweet, vanilla‐like fragrance but is subject to regulatory attention due to potential hepatotoxic effects at high intake levels [38].

In the current study, the cinnamaldehyde‐to‐coumarin ratio was approximately 30.7. This finding is in agreement with previous metabolomic studies [37, 39], supporting the characteristic chemical profile of the oil and distinguishing it from *Cinnamomum zeylanicum*, which generally contains substantially lower levels of coumarin. (Z)‐cinnamaldehyde, present at 1.26%, contributes a milder aromatic profile due to its cis configuration, while (Z)‐2‐methoxycinnamaldehyde (2.2%), an aromatic aldehyde with a methoxy substituent, provides subtle almond‐like notes, and is associated with the overall aroma complexity [35].

(+)‐2‐bornanone was present at a concentration of 1.91%; it is a bicyclic monoterpene ketone that may influence the overall sensory profile of CEO. The least intense peak in the chromatogram corresponded to hydrocinnamaldehyde (0.77%), which imparts sweet and woody nuances. Collectively, these minor components may enhance the chemical and sensory complexity of the essential oil [40].

It is important to note that the composition of *Cinnamomum cassia* essential oil is highly dynamic and subject to natural variations. A range of factors, including the geographical origin of the bark, climate, soil quality, seasonal harvesting time, plant age, and extraction parameters can alter the relative abundance of individual phytoconstituents. Consequently, while (E)‐cinnamaldehyde remains a major characteristic constituent, the percentages of minor compounds such as coumarin, cinnamyl acetate, and methoxycinnamaldehyde may vary considerably between different botanical batches. Such variations in the chemical profile can contribute to differences in the biological activities reported for the oil [32, 41].

### 3.3 Antibacterial Activity and Determination of MIC and MBC

**FIGURE 3:**
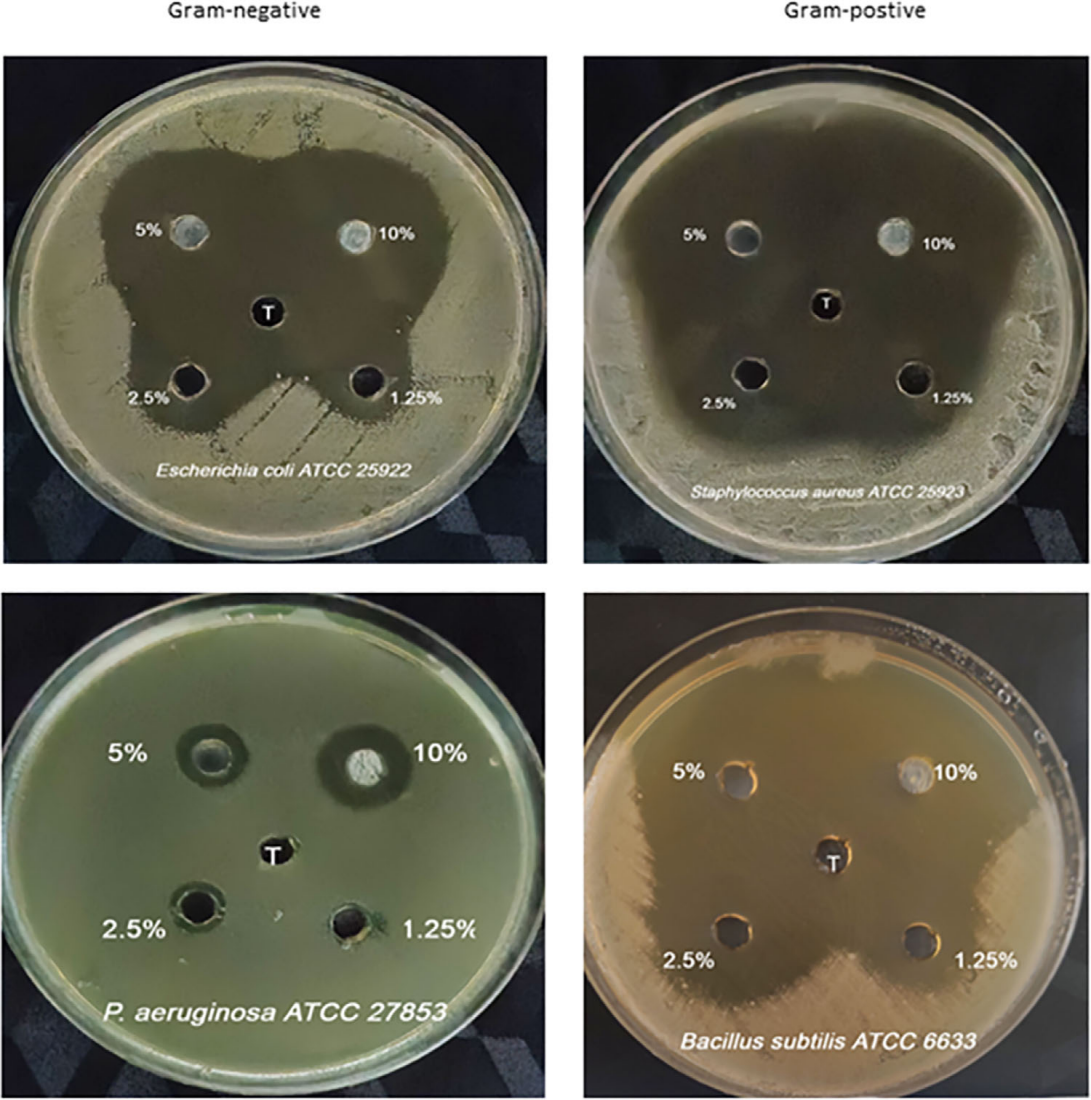
Representative photographs of the antibacterial activity of *Cinnamomum cassia* essential oil (CEO) against the tested bacterial strains at different concentrations (10%, 5%, 2.5%, and 1.25%, v/v) using the agar well diffusion assay. DMSO was used as the negative control (original photographs by the authors).

*Cinnamomum cassia* essential oil exhibited concentration‐dependent antibacterial activity against the tested gram‐positive and gram‐negative bacteria, with inhibition generally decreasing as the CEO concentration decreased (Tables 2, 3 and 4; Figure 3).

**TABLE 2:** Inhibition zones (mm) of *Cinnamomum cassia* essential oil (CEO) at different concentrations against the tested bacterial strains.

Strains	Inhibition zones (mm) Concentration of CEO (%)			
	10%	5%	2.5%	1.25%
*S. aureus* ATCC 25923	47.00 ± 1.00^a^	38.00 ± 1.04^b^	30.00 ± 00^c^	19.00 ± 1.00^d^
*E. coli* ATCC 25922	31.33 ± 0.57^a^	27.00 ± 3.00^a^	19.66 ± 1.52^b^	14.33 ± 1.52^c^
*P. aeruginosa* ATCC 27853	16.00 ± 1.00^a^	11.66 ± 0.57^b^	9.00 ± 1.00^c^	—
*B. subtilis* ATCC 6633	45.00 ± 1.00^a^	37.00 ± 1.15^b^	27.00 ± 2.08^c^	18.33 ± 1.52^d^

Values are expressed as means ± SD (*n* = 3). Different superscript lowercase letters within the same bacterial strain indicate significant differences (*p *< 0.05) among CEO concentrations, as determined by one‐way analysis of variance (ANOVA) followed by Tukey's post hoc test. “–” indicates no inhibition zone. The DMSO negative control showed no inhibition zone against any of the tested bacterial strains.

**TABLE 3:** Minimum inhibitory concentrations (MICs) and minimum bactericidal concentrations (MBCs) (mg/mL) of CEO against the selected bacterial strains.

Strains	MIC (mg/mL)	MBC (mg/mL)
*S. aureus* ATCC 25923	0.52	2.08
*E. coli* ATCC 25922	1.04	1.04
*P. aeruginosa* ATCC 27853	1.04	1.04
*B. subtilis* ATCC 6633	0.52	0.52

**TABLE 4:** Molecular docking scores and predicted binding affinities of CEO constituents against selected bacterial and fungal protein targets.

Target		(+)‐2‐Bornanone		(E)‐Cinnamaldehyde		(Z)‐Cinnamaldehyde		Cinnamyl acetate		Coumarin		Hydrocinnamaldehyde		Methoxycinnamaldehyde	
	PDB ID	Energy (kcal/mol)	Ki (µM)	Energy (kcal/mol)	Ki (µM)	Energy (kcal/mol)	Ki (µM)	Energy (kcal/mol)	Ki (µM)	Energy (kcal/mol)	Ki (µM)	Energy (kcal/mol)	Ki (µM)	Energy (kcal/mol)	Ki (µM)
**TEM‐1 β‐lactamase**	**1NYM**	−6	39.99	−6.2	28.53	−6.3	24.10	−6.7	12.27	−7.7	2.27	−6.2	28.53	−6,7	12.27
**D‐alanine:D‐alanine ligase**	**2ZDQ**	−5.4	110.10	−6.7	12.27	−6.7	12.27	−7.4	3.76	−7.7	2.27	−6.4	20.36	−6,9	8.76
**penicillin‐binding protein 1a**	**3UDI**	−5.6	78.55	−5	216.26	−4.9	256.02	−5.4	110.10	−5.9	47.34	−4.7	358.82	−4,8	303.09
**Dihydropteroate synthase**	**2VEG**	−6.1	33.78	−5.4	110.10	−5.4	110.10	−6.2	28.53	−6.2	28.53	−6	39.99	−5,1	182.67
**Dihydrofolate reductase**	**3SRW**	−4.3	704.82	−4.4	595.36	−4.3	704.82	−4.9	256.02	−4.9	256.02	−4	1169.44	−4,5	502.89
**DNA gyrase**	**1KZN**	−4.6	424.79	−5.2	154.30	−5.4	110.10	−6	39.99	−6.6	14.53	−5.3	130.34	−5,5	93.00
**IV topoisomerase**	**3RAE**	−4.8	303.09	−5	216.26	−5.1	182.67	−5.7	66.35	−6.1	33.78	−4.6	424.79	−5,6	78.55
**tyrosyl‐tRNA synthetase**	**1 × 8X**	−5.5	93.00	−5.9	47.34	−5.5	93.00	−6.3	24.10	−7	7.40	−5.3	130.34	−5,6	78.55
**Isoleucyl‐tRNA synthetase**	**1JZQ**	−6	39.99	−5.7	66.35	−5.6	78.55	−5.9	47.34	−6.3	24.10	−5.3	130.34	−5,8	56.05
**Aspergillus niger epoxide hydrolase**	**3G0I**	−6.6	14.53	−5.7	66.35	−6	39.99	−6.3	24.10	−6.8	10.36	−5.5	93.00	−6	39.99
**sterol 14‐alpha demethylase (CYP51B)**	**5FRB**	−6.2	28.53	−6	39.99	−5.9	47.34	−6.7	12.27	−6.9	8.76	−5.7	66.35	−6	39.99

Overall, the gram‐positive strains appeared more susceptible than the gram‐negative strains. This pattern is consistent with previous observations for *C. cassia* essential oil, although the magnitude of susceptibility can vary substantially among bacterial species. Nguyen et al. [42] similarly reported greater susceptibility of gram‐positive bacteria to *C. cassia* oil and suggested that differences in cell‐envelope structure and membrane permeability may contribute to this variation. Their study also showed that the activity of the whole essential oil could differ from that of isolated (E)‐cinnamaldehyde, supporting the view that the antimicrobial effect of CEO cannot necessarily be attributed to its major constituent alone [42].

The MIC/MBC profile further supported the greater susceptibility of the gram‐positive strains. Compared with the values of Huang et al. [43] (MICs: 2.5–10 mg/mL; MBCs: 5–20 mg/mL), the lower MIC and MBC values obtained indicate that the tested strains were more sensitive under the current experimental conditions, likely reflecting variations in strain susceptibility, assay methodology, or essential oil composition. Based on the MBC/MIC ratios (1–4), the tested CEO showed a bactericidal effect against all four bacterial strains under the present experimental conditions [44]. This activity may be partly associated with the high abundance of (E)‐cinnamaldehyde (87.14%) in the tested CEO, as cinnamaldehyde has been reported to disrupt bacterial membrane integrity and interfere with cellular energy metabolism, including the proton motive force and ATP generation [45, 46]. However, the contribution of other identified constituents cannot be excluded, and the individual or combined effects of the CEO constituents were not experimentally evaluated in the present study.

### 3.4 Antifungal Activity and Determination of MIC and MFC of CEO

**FIGURE 4:**
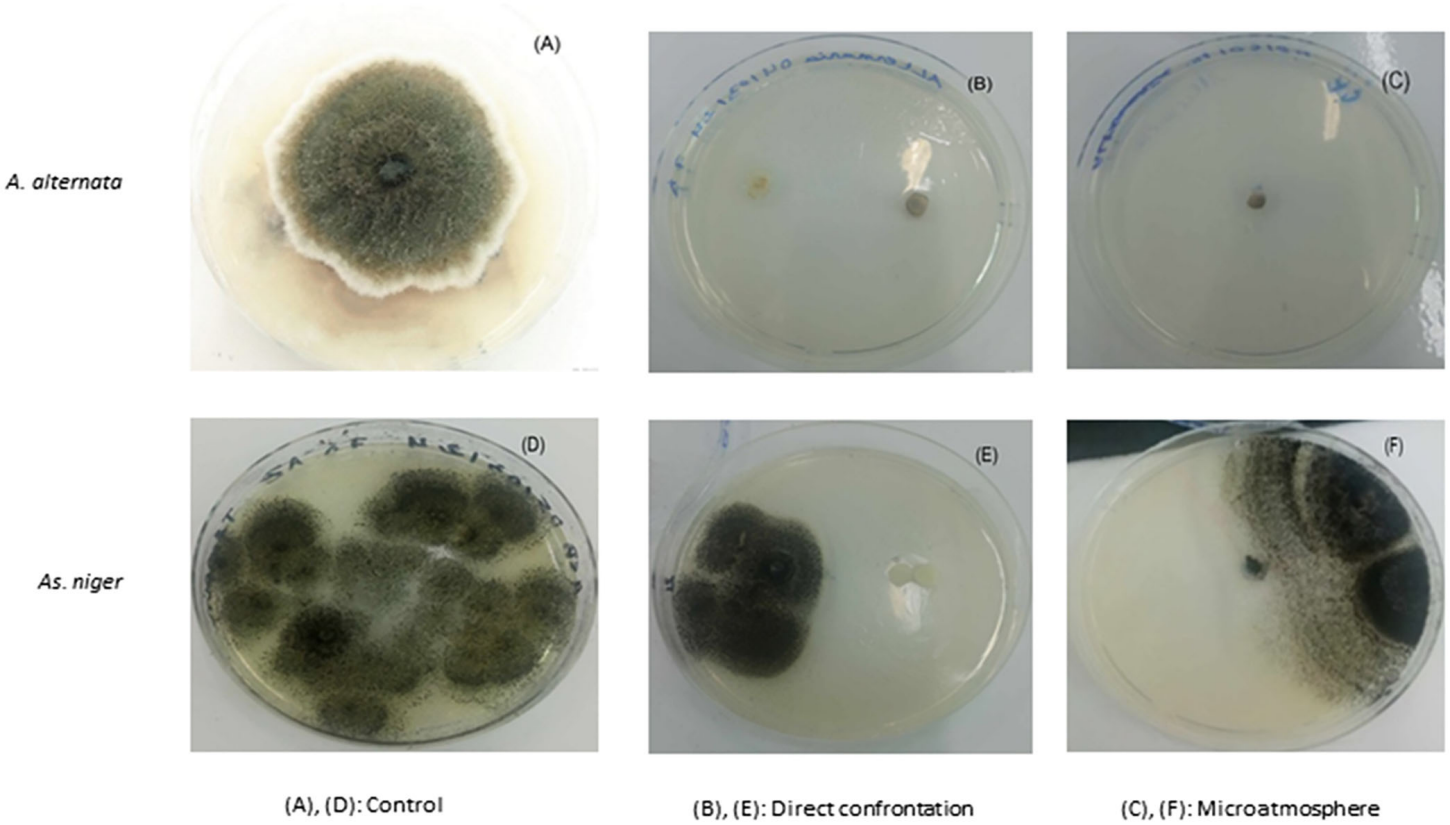
Representative photographs illustrating the antifungal activity of *Cinnamomum cassia* essential oil (CEO) against the tested fungal strains using the direct confrontation and microatmosphere methods (original photographs by the authors).

Cinnamomum cassia essential oil (CEO) demonstrated antifungal activity against both *Alternaria alternata* and *Aspergillus niger* (Figure 4). In direct contact assays, the oil completely inhibited *A. alternata* growth, while *As. niger* showed 79.18% ± 0.12% inhibition. In the microatmosphere assay, *A. alternata* remained fully suppressed, whereas *As. niger* inhibition decreased to 41.46% ± 0.14%. The high inhibition observed under direct‐contact conditions, particularly for *As. niger*, indicates that the mode of exposure influenced the antifungal effectiveness of CEO.

This difference may reflect the greater availability of CEO constituents to the fungal biomass under direct‐contact conditions compared with the vapor phase, particularly against *As. niger*. These findings are consistent with previous reports indicating that cinnamon oil is highly effective against *A. alternata* [47] and moderately effective against *As. niger* [48]. The negative control plates showed normal mycelial growth without detectable inhibition for both *A. alternata* and *As. niger*, confirming that the observed antifungal effects were attributable to CEO.

The minimum inhibitory concentrations (MICs) of CEO were <0.5 µL/mL for *A. alternata* and 2 µL/mL for *As. niger*, with corresponding minimum fungicidal concentrations (MFCs) of 1 and 16 µL/mL, respectively. The MFC/MIC ratio was ≤2 for *A. alternata*, indicating a fungicidal effect, whereas a ratio of 8 was observed for *As. niger*, indicating a predominantly fungistatic effect. These results indicate species‐specific susceptibility to CEO, with *A. alternata* showing greater susceptibility than *As. niger* under the conditions tested. Lower MIC values against *As. niger* (25 µg/mL) have been reported in a previous study [49]; however, *C. cassia* oil demonstrated higher efficacy against *A. alternata* than *C. zeylanicum* oil [50].

The antifungal activity of CEO may be partly attributed to its high content of (E)‐cinnamaldehyde (87.14%). Previous studies have suggested that cinnamaldehyde can interfere with fungal cell wall and membrane functions, including β‐(1, 3)‐glucan and chitin synthesis, as well as ATPase activity. Minor constituents, including (Z)‐cinnamaldehyde (1.26%), (Z)‐2‐methoxycinnamaldehyde (2.2%), cinnamyl acetate (3.87%), and coumarin (2.84%) may also contribute in the overall activity. Mechanistic studies have further indicated that oil exposure increases alkaline phosphatase activity and electrolyte leakage in fungal cultures, reflecting cell wall and membrane damage and loss of membrane permeability control [49, 51]. However, these mechanisms were not directly investigated in this study and remain to be confirmed experimentally. Moreover, the antimicrobial activity observed under the present in vitro conditions may differ in real food systems, where interactions with food matrix components can affect the activity of essential oil constituents [52].

### 3.5 Docking Study

Molecular docking was conducted as a predictive computational analysis to explore the potential interactions of the identified CEO phytochemicals with selected bacterial and fungal targets.

The antibacterial potential of the selected compounds was evaluated using molecular docking simulations against several key bacterial target proteins. Binding energies (expressed in kcal/mol) and calculated inhibition constants (Ki, in µM) were used to assess the relative binding affinity of each compound. Among the tested compounds, coumarin, followed by cinnamyl acetate, showed the most favourable predicted binding affinities across all studied targets, with a maximum predicted binding energy of −7.7 kcal/mol and an inhibition constant of 2.27 µM against TEM‐1 β‐lactamase (1NYM) and D‐alanine:D‐alanine ligase (2ZDQ), enzymes that play essential roles in bacterial cell wall synthesis. Moreover, coumarin showed a predicted binding energy of −7.0 kcal/mol against tyrosyl‐tRNA synthetase (1 × 8X), an enzyme involved in bacterial protein synthesis, indicating favorable predicted ligand–enzyme interactions.

**FIGURE 5:**
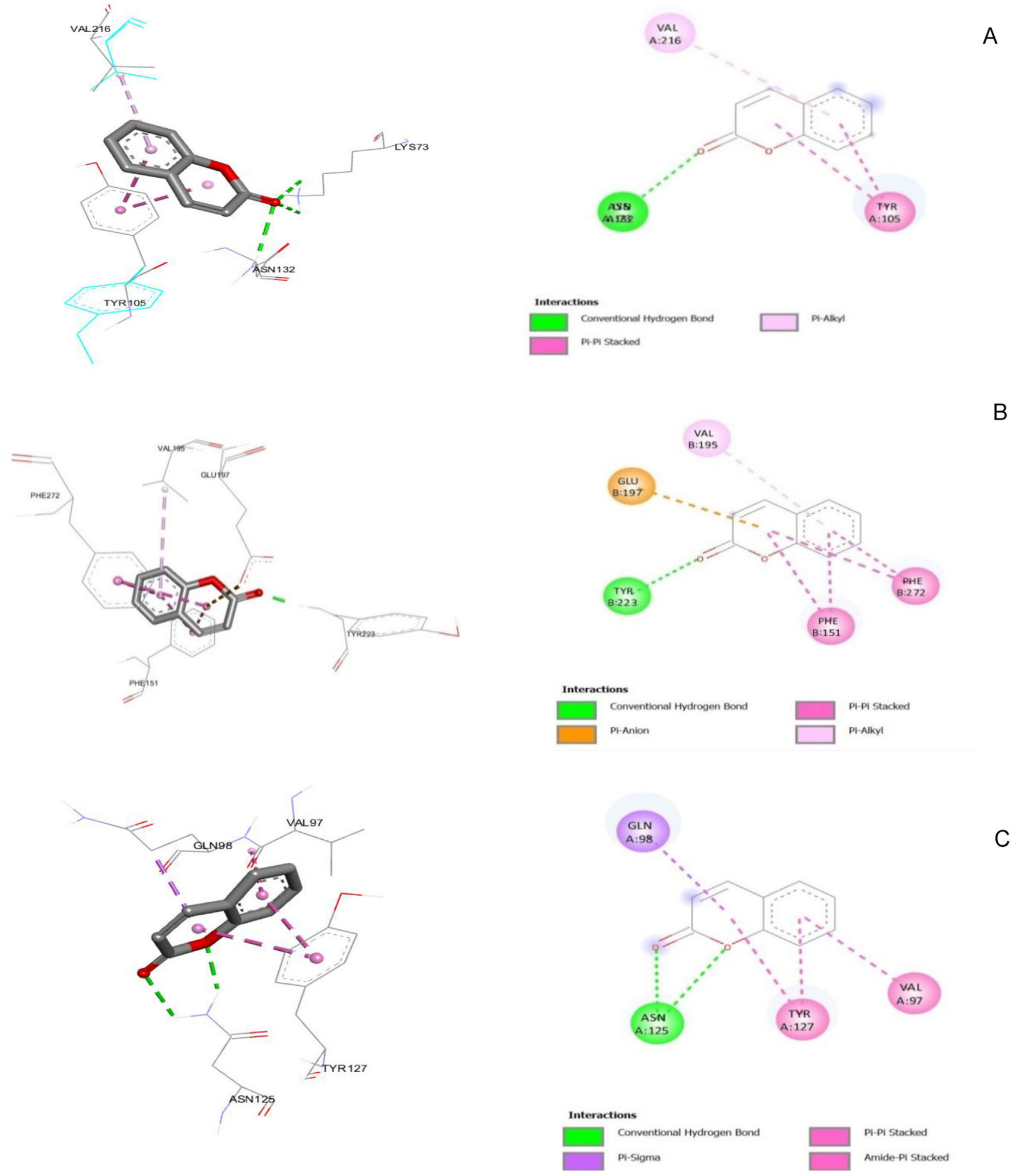
Interactions of coumarin with TEM‐1 β‐lactamase (A), D‐alanine:D‐alanine ligase (B), tyrosyl‐tRNA synthetase (C) (2D and 3D).

Similarly, molecular docking results revealed that coumarin showed a favourable predicted binding affinity for sterol 14‐α‐demethylase (CYP51B) (PDB: 5FRB) and epoxide hydrolase (EH) from *Aspergillus niger* (PDB: 3G0I) with predicted binding energies of −6.9 and −6.8 kcal/mol (Ki = 8.76 and 10.36 µM, respectively). These findings suggest that coumarin may interact with multiple fungal targets involved in essential cellular processes, potentially contributing to its observed antifungal activity. Molecular docking results revealed that coumarin displayed a strong predicted binding affinity toward three target enzymes: TEM‐1 β‐lactamase (PDB ID: 1NYM), D‐alanine:D‐alanine ligase (PDB ID: 2ZDQ), and tyrosyl‐tRNA synthetase (PDB ID: 1 × 8X) (Table 5, Figure 5). The highest predicted binding energy (−7.7 kcal mol^−1^) was observed with TEM‐1, primarily attributed to three hydrogen bonds formed by coumarin acting as a hydrogen donor, together with π–π stacking and π–alkyl interactions that may enhanced complex stability. In the D‐alanine:D‐alanine ligase (2ZDQ) complex, the ligand was stabilized through four π–π stacking interactions involving residues Phe151 and Phe272, in addition to one π–alkyl contact. Similarly, the 1 × 8X complex exhibited two hydrogen bonds, two π–π stacking, one π–σ, and one amide–π stacking interaction, indicating favorable ligand–enzyme interaction (Table 5, Figure 5). These results suggest that coumarin possesses favorable molecular recognition properties that may contribute to its antimicrobial activity, as coumarin and its derivatives are recognized as promising bioactive compounds with a broad spectrum of antimicrobial effects, particularly against antibiotic‐resistant bacterial strains. Numerous recent in vitro and in silico studies have further highlighted their efficacy, diverse mechanisms of action, and potential as novel antibacterial agents.

**TABLE 5:** Number and types of interactions involved in the binding of coumarin to selected protein targets identified by molecular docking.

Target		Hydrogen bond	Amide‐Pi Stacked	Pi‐ alkyl	Pi‐stacked	Pi‐anion	Pi‐sigma
Interactions of coumarin with TEM‐1 β‐lactamase1NYM	Number	3	/	1	2		/
	Amino acid	LYS:73 LYS:73 ASN:132	/	VAL:216	TYR:105 TYR:105		/
Interactions of coumarin with D‐alanine:D‐alanine ligase (2ZDQ)	Number	1	/	1	4	1	/
	Amino acid	/	/	LEU: 192	PHE: 151 PHE: 151 PHE: 272 PHE: 272	GLU:197	/
Interactions of coumarin with tyrosyl‐tRNA synthetase (1 × 8X)	Number	2	1	/	2		1
	Amino acid	ASN A: 125 ASN A: 125	VAL97:C,O;GLN98	/	TYR A: 125 TYR A: 125		GLN A: 98

The antibacterial activity of coumarin has been associated with their interactions with key bacterial enzymes and proteins. For instance, Latif et al. [53] reported that ligand L3 exhibited a high predicted binding affinity (−39.92 kcal/mol) against *Streptococcus pyogenes*, surpassing that of the reference antibiotic, likely due to its capacity to form multiple hydrogen bonds. Beyond enzyme inhibition, coumarin derivatives have demonstrated additional antimicrobial mechanisms. Ungureanu et al. [54] showed that coumarin–thiazole hybrids possess significant antibacterial and antibiofilm activities, particularly against *Pseudomonas aeruginosa* and *Enterococcus faecalis*, with stable interactions observed at the GyrB subunit of DNA gyrase.

Coumarins have also been reported to target bacterial resistance mechanisms. Abdel‐Halim et al. [55] demonstrated that coumarin inhibited carbapenemase expression (NDM‐1, VIM‐2, OXA‐9) in *Klebsiella pneumoniae*, restored the efficacy of meropenem, and exhibited predicted binding energies ranging from −6.2 to −7.8 kcal/mol. In addition, coumarin derivatives can interfere with quorum sensing (QS) systems and biofilm formation, which are critical for bacterial virulence. Qais et al. [56] reported inhibition of QS‐regulated virulence factors in *P. aeruginosa* and *Serratia marcescens*, supported by favorable strong docking scores (−5.7 to −8.1 kcal/mol) and stable molecular dynamics profiles. Furthermore, virtual screening studies have identified coumarin‐based hybrids with promising antibacterial potential; for example, Mishra et al. [57] highlighted coumarin‐1,2,3‐triazole hybrid 176 as a strong candidate based on its binding stability and favorable ADMET properties.

Recent in silico investigations have also highlighted the antifungal potential of coumarin‐based compounds through molecular docking, molecular dynamics simulations, and structure–activity relationship (SAR) analyses. Nuha et al. [58] synthesized a series of coumarin derivatives, among which compound 4e exhibited potent antifungal activity (MIC = 0.97 µg/mL) against *Candida parapsilosis*, outperforming standard antifungals such as voriconazole and fluconazole. Docking and simulation studies confirmed strong interactions of compounds 4e and 4a with key fungal targets, including CYP51 and thymidylate synthase (TS). Similarly, Sabt et al. [59] reported coumarin derivatives with high binding affinity (−12.0 kcal/mol) toward CYP51, supported by multiple hydrogen bond interactions within the active site. More recently, Ungureanu et al. (2025) [54] identified hydroxyphenyl‐thiazolyl‐coumarin hybrids with potent antifungal and antibiofilm activities, showing stronger binding affinities than fluconazole and notable activity against *Aspergillus brasiliensis* (MIC = 15.62 µg/mL). These findings consistently identify CYP51 as a potential molecular target of coumarin‐based compounds, supporting the possibility that the predicted interaction of coumarin with CYP51 may contribute to the antifungal activity observed in the present study.

These docking results provide potential mechanistic insights into the antimicrobial activity of CEO by suggesting that coumarin and cinnamyl acetate may interact with bacterial targets involved in β‐lactam resistance, cell wall biosynthesis, and protein synthesis. In fungi, coumarin showed predicted interactions with CYP51B and epoxide hydrolase, suggesting potential effects on sterol biosynthesis and cellular homeostasis. These findings provide plausible molecular targets underlying the observed antimicrobial activity and generate testable mechanistic hypotheses. However, docking predictions do not establish target inhibition or confirm the antimicrobial mechanism experimentally.

## 4 Conclusion

*Cinnamomum cassia* essential oil exhibited antibacterial and antifungal activity, with differences in susceptibility among the bacterial and fungal strains tested. The antibacterial activity was particularly pronounced against the gram‐positive strains, while *Alternaria alternata* showed high susceptibility to CEO. Molecular docking further revealed favorable predicted binding interactions of coumarin with selected bacterial and fungal targets, providing potential molecular‐level insights into the antimicrobial activity of CEO. However, these computational findings remain predictive and require experimental validation. Further studies should investigate the effectiveness and stability of CEO in real food systems to assess its potential application as a natural biopreservative.

## Author Contributions

**Asma Bouguerra**: writing – original draft, visualization, methodology, investigation, formal analysis, conceptualization. **Asma Meziti**: investigation, data curation. **Hassina Guergour**: investigation, funding acquisition, data curation. **Naouel Boussoualim**: methodology, formal analysis, conceptualization. **Imane Krache**: formal analysis, data curation, writing – review and editing. **Nadira Oukala**: writing – review and editing. **Amal Saidi**: writing – review and editing. **Daoud Harzallah**: writing – review and editing and supervision. **Hamdi Bendif**: funding acquisition, writing – review and editing and supervision. **Gharieb S. El‐Sayyad and Shakilur Rahman**: funding acquisition, writing – review and editing. **Stefania Garzoli**: writing – review and editing and supervision.

## Conflicts of Interest

The authors declare that they have no known competing financial interests or personal relationships that could have appeared to influence the work reported in this paper.

## Data Availability

All data will be available upon request from the authors.
